# Mala flavor preference increases risk of excessive gestational weight gain mediated by high-carbohydrate dietary patterns in Chongqing, China: an ambispective cohort study

**DOI:** 10.3389/fnut.2024.1464748

**Published:** 2025-01-07

**Authors:** Jinghua Li, Difei Wang, Yanyan Mao, Wuxia Zhang, Qianxi Zhu, Jun Liu, Jing Du, Weijin Zhou, Fen Wang, Min Li

**Affiliations:** ^1^Kaizhou District Maternal and Child Health Hospital, Chongqing, China; ^2^Shanghai-MOST Key Laboratory of Health and Disease Genomics, NHC Key Lab of Reproduction Regulation, Shanghai Institute for Biomedical and Pharmaceutical Technologies, Shanghai, China; ^3^NHC Key Laboratory of Birth Defects and Reproductive Health (Chongqing Population and Family Planning Science and Technology Research Institute), Chongqing, China

**Keywords:** pregnant women, dietary patterns, taste preference, gestational weight gain, mediation analysis

## Abstract

Taste preference drives food selection, acceptance, or rejection and influences nutritional status and body mass index. Nevertheless, there are few reports concerning pregnant women. Mala flavor, characterized by its “numbing” and “spicy” sensations, is a distinctive taste of Sichuan cuisine, created by the combination of Chinese prickly ash and chili peppers. We conducted a cohort study in Chongqing, China to analyze the impact of Mala flavor, on excessive gestational weight gain (GWG). The study included 495 pregnant women aged 20–45 years, without chronic diseases, who conceived naturally and had single pregnancies from May 2021 to November 2022. Demographic information and pregnancy outcomes were collected during the second trimester and post-delivery, respectively. Food intake and taste preferences, including fatty, salty, and Mala flavors, were assessed during the third trimester. Latent Profile Analysis revealed three dietary patterns: “high-carbohydrate diet” (HCD), “low-carbohydrate diet” (LND), and “moderate nutrient diet” (MND). Multiple logistic regression indicated that pregnant women preferring Mala flavor were more likely to follow an HCD and had a higher risk of excessive GWG. Moreover, those adhering to an HCD were at an increased risk of excessive GWG. Mediation analysis showed that the preference for Mala flavor influenced excessive GWG through HCDs, with a significant indirect effect and an insignificant direct effect. Our study suggests that a preference for Mala flavor is positively associated with excessive GWG, mediated by HCD patterns. However, these findings should be approached with caution due to the exploratory nature of the study.

## 1 Introduction

The “Developmental Origins of Health and Disease” theory posits that early-life nutrition is a key determinant in the development of chronic conditions such as hypertension, diabetes, and cardiovascular disease in adulthood (1). Gestational weight gain (GWG) serves as a direct measure of maternal nutrition and is an independent predictor of both perinatal and long-term health outcomes for both the mother and the infant (2). Studies show that inadequate GWG can raise the risk of infants being small for gestational age and having low birth weight, while excessive GWG may precipitate conditions like pregnancy-induced hypertension syndrome, preeclampsia, gestational diabetes mellitus, large for gestational age, and an increased need for cesarean section (3–5). Furthermore, excessive GWG can have lasting health repercussions, predisposing women to diabetes and cardiovascular diseases later in life and increasing the risk of childhood obesity in their offspring (6–8).

Nutrition during pregnancy is crucial, as it impacts not only the mother’s health but also profoundly influences the growth and development of the fetus (9). A balanced and well-rounded diet is fundamental to controlling GWG and preventing pregnancy-related diseases (10). Analyzing dietary patterns, rather than individual foods or nutrients, provides a more effective approach to evaluating overall dietary status (11). There are various methods to assess dietary patterns: Priori methods, are based on existing dietary guidelines or scientific dietary recommendations and use scores to reflect the degree of adherence, such as index or score analysis (12, 13). Posteriori methods, aim to identify factors that explain variations in dietary patterns or classify individuals into distinct groups. Examples include factor analysis, cluster analysis, and latent profile analysis (LPA) (14–16).

The variations in geography, climate, history, customs, and assessment methods contribute to the diversity in reported dietary patterns across studies. Consequently, the impact of these patterns on GWG is a subject of inconsistent findings. A recent Danish study highlighted the advantages of a high-protein, low-glycemic index diet, particularly in reducing GWG and associated complications, such as a decreased rate cesarean deliveries among overweight or obese women (17). A retrospective study identified a negative association between GWG and adherence to the Mediterranean dietary pattern, which is characterized by a high consumption of vegetables, whole cereals, nuts, fish, and olive oil and a low consumption of refined cereals, snacks, and desserts (18). Additionally, a prospective cohort study from central China revealed that a dietary pattern rich in beans and vegetables is beneficial for managing GWG effectively and for promoting higher birth weight in newborns (19).

Recent studies have sought to explore the potential correlations between specific tastes preference, dietary habits, and obesity. A systematic review, which meticulously examined 19 articles, revealed a possible association between obesity and taste preferences, particularly a moderate inclination toward salty, bitter, and fatty flavors, as well as a heightened preference for sour flavors (20). However, there remains a significant gap in the literature concerning the relationship between taste preference and GWG.

In Chongqing, southwest China, the distinctive Mala flavor is a cornerstone of local cuisine. This flavor, characterized by its “numbing” and “spicy” qualities, is typically achieved by the addition of Chinese prickly ash, known for its tingling effect, and chili peppers to a variety of dishes including fish, poultry, meat, and soy products (21). Originating from Sichuan cuisine, the Mala taste has transcended regional boundaries, becoming a beloved seasoning across China and gaining international recognition for its unique sensory experience. However, the impact of this flavor profile on body weight remains largely unexplored in scientific literature. Furthermore, research on the connection between diet and GWG in Chongqing is scarce, which poses a challenge for the development of tailored nutritional intervention. In light of these gaps, our study aims to investigate the correlation between dietary habits, including taste preferences and dietary patterns, and GWG among pregnant women in Chongqing.

## 2 Materials and methods

### 2.1 Study population

This ambispective cohort study was conducted at Chongqing Kaizhou Maternal and Child Health Hospital. Pregnant women aged 20–45 years who underwent routine prenatal care at the hospital obstetrics department at 24–27 gestational weeks and were expected to deliver at the hospital were invited to enroll in the cohort from May 2021 to November 2022. Women who had undergone assisted reproductive technology (ART; e.g., ovulation induction, *in vitro* fertilization, intracytoplasmic sperm injection treatment, or intrauterine insemination); had chronic medical and surgical diseases or pregnancy complications; were confirmed to have multiple pregnancies; or had cognitive impairment, dementia, or severe mental illness were excluded from this study.

The ethics committee of Chongqing Kaizhou Maternal and Child Health Hospital approved this study. Written informed consent was obtained from all participants.

### 2.2 Measures

The participants were interviewed by trained obstetric nurses three times: at enrollment (24–27 gestational weeks), during the third trimester (32–36 gestational weeks), and after the delivery. Demographic information was collected at the time of enrollment. Data on dietary status, including taste preference and food intake over the previous month, were collected at 32–36 gestational weeks. Pregnancy outcomes were recorded after the delivery. GWG was calculated by subtracting the pre-pregnancy weight from the weight at the end of pregnancy. Inadequate or excessive GWG were defined, respectively, as GWG not meeting or exceeding the recommended GWG for each pre-pregnancy BMI category by gestational age at delivery on the basis of the Institute of Medicine guidelines (IOM) (22). Taste preference—fatty, salty, or Mala flavor—was recorded. The degree of preference was categorized as no or light, medium, or heavy. A streamlined and improved version of food frequency questionnaire (FFQ), building upon the 2014 edition (23), was utilized to collect data on dietary intake during pregnancy. The FFQ comprises 15 food items covering nine distinct categories, namely, cereals, vegetables, fruits, beans, meat, poultry, aquatic products, eggs, and dairy. For each food item in the FFQ, participants reported the frequency of consumption and portion size.

The sufficiency and quality of food consumption were determined using the Chinese Balanced Dietary Pagoda (CBDP) 2022 version for pregnant women (Supplementary Figure 1). The CBDPs, established and updated by the Chinese Nutrition Association, are visual representations of dietary guidelines for special groups of Chinese residents, including average adults, children, adolescents and pregnant women (24). These guidelines are crafted in accordance with principles of nutritional science and take into account the unique aspects of the Chinese dietary structure. CBDPs is structured into five distinct levels, each with a diminishing size from the base to the pinnacle. This hierarchy corresponds to the recommended daily consumption levels for five key food groups: cereals, vegetables and fruits, animal-based foods, legumes and nuts, and finally, cooking oils and salt.

### 2.3 Statistical analyses

Participants’ characteristics, taste preferences, and daily food intakes were presented as mean ± standard deviation for continuous variables and as frequencies with proportions for categorical variables. Differences in variables between groups were evaluated using an analysis of variance (ANOVA) for continuous variables, and Pearson Chi-square tests for classification. Owing to low frequency, heavy taste preference was merged into the medium category for analysis.

LPA was used to identify mutually exclusive dietary patterns. The quartiles of daily food intake were used as exogenous variables. The optimal number of latent profiles was determined comprehensively by evaluating the goodness of fit of each model according to Akaike Information Criteria (AIC), Bayesian Information Criterion (BIC), and adjusted BIC (aBIC), as well as Entropy, Bootstrap Likelihood Ratio test (BLRT), and Lo–Mendell–Rubin likelihood ratio test (LMR) (25). Lower statistical values of AIC, BIC, and aBIC indicated a better fit. Entropy values range from 0 to 1, where a value closer to 1 indicates a more accurate classification. If *p* < 0.05 for LMR or BLRT, the k-category model is superior to the k-1 category model (25).

The association between taste preference, dietary pattern, and excess GWG was analyzed using a multiple logistic regression model, and adjustments were conducted for potential confounders that were selected on the basis of ANOVA. The mediating effect of dietary patterns on the relationship between taste preference and excess GWG was examined using mediation analysis.

LPA and mediation analysis were conducted using Mplus version 7.4. The rest of the analyses were performed using SPSS version 21.0. There was one missing value for BMI, which was replaced with the average value in the relative analysis.

## 3 Results

### 3.1 Demography of the pregnant women

Among the 635 pregnant women screened, 135 were excluded for intending to deliver at a different hospital (*n* = 3), undergoing ART (*n* = 1), having multiple pregnancies (*n* = 2), and having chronic medical disease (*n* = 129). Of the remaining 500 pregnant women, three were lost to follow-up, and two had stillbirths. Therefore, a final sample of 495 women was analyzed.

The pregnant women in this study had an average age of 27.8 (±4.4) years, with a mean pre-pregnancy body mass index (BMI) of 21.4 (± 0.7 kg/m^2^). More than one-third were first-time pregnancies (36.0%), and the majority were from agricultural households (73.9%). Additionally, more than half had a junior high school education or below (51.3%), and the majority did not hold official employment roles (71.9%).

The pregnant women gained an average of 14.0 (±4.9) kg throughout their pregnancy. Of the total participants, 23.4 and 30.3% had inadequate and excessive GWG, respectively. Table 1 presents the demographic characteristics of pregnant women according to GWG. We noted only one significant difference in pre-pregnancy BMI among the three GWG groups (*p* < 0.01).

**Table 1:** Demographic characteristics of pregnant women in Chongqing according to gestational weight gain.

Variations	Total population	Gestational weight gain, *n* (%)			*p*
		Inadequate	Adequate	Excessive	
*N*	495	116	229	150	
Age, y					0.326
~25	136 (27.5)	36 (31.0)	63 (27.5)	37 (24.7)	
~30	222 (44.8)	46 (39.7)	111 (48.5)	65 (43.3)	
>30	137 (27.7)	34 (29.3)	55 (24.0)	46 (32.0)	
Pre-pregnancy BMI, kg/m^2^					< 0.001
<18.5	55 (11.1)	11 (9.5)	37 (16.2)	7 (4.7)	
18.5 ~ 23.9	359 (72.5)	80 (69.0)	171 (74.7)	108 (72.0)	
≥24	81 (16.4)	25 (21.6)	21 (9.2)	35 (23.3)	
Gravidity					0.331
1	178 (36.0)	35 (30.2)	86 (37.6)	57 (38.0)	
>1	317 (64.0)	81 (69.8)	143 (62.4)	93 (62.0)	
Place of residence					0.393
Agricultural	366 (73.9)	91 (78.4)	164 (71.6)	111 (74.0)	
Non-Agricultural	129 (26.1)	25 (21.6)	65 (28.4)	39 (26.0)	
Educational qualifications					0.611
Junior high school or below	254 (51.3)	60 (51.7)	116 (50.7)	78 (52.0)	
Senior high school	141 (28.5)	38 (32.8)	63 (27.5)	40 (26.7)	
University and above	100 (20.2)	18 (15.5)	50 (21.8)	32 (21.3)	
Occupation					
Farmer	24 (4.8)	9 (7.8)	6 (2.6)	9 (6.0)	0.070
Business/working	42 (8.5)	5 (4.3)	26 (11.4)	11 (7.3)	
Teachers/civil servants/staff	55 (11.1)	9 (7.8)	29 (12.7)	17 (11.3)	
Unemployed	356 (71.9)	86 (74.1)	160 (69.9)	110 (73.3)	
Others	18 (3.6)	7 (6.0)	8 (3.5)	3 (2.0)	

BMI: body mass index.

### 3.2 Dietary status of pregnant women

Most of the pregnant women had medium taste preferences for fatty, and salty flavors (82.2, and 77.6%, respectively) and had no or light taste preferences for Mala flavors (73.7%).

Their daily intake of cereals, meat, poultry, and eggs approached or exceeded the recommended levels. Their intake of vegetables, beans, and aquatic products fell significantly short, with less than 60% of the pregnant women meeting the minimum recommended intake for these food categories (Table 2).

**Table 2:** Daily food category intakes of pregnant women in Chongqing.

Food categories	Recommended dietary intakes (g)	Mean ± SD	
		Actual dietary intakes (g)	Relative intake (%)^a^
Cereals	225–275	214.4 ± 108.5	95 ± 49
Vegetables	400–500	83.4 ± 70.1	21 ± 18
Fruits	200–350	129.0 ± 111.3	65 ± 56
Beans	20	10.5 ± 13.6	53 ± 68
Meat and Poultry	50–75	43.9 ± 47.4	129 ± 112
Aquatic products	75–100	12.8 ± 17.3	17 ± 23
Eggs	50	50.2 ± 27.0	101 ± 54
Dairy	300–500	205.6 ± 93.2	69 ± 29

^aRelative intake was calculated as a percentage of the minimum recommended intake.^

SD, standard deviation.

**Figure 1:**
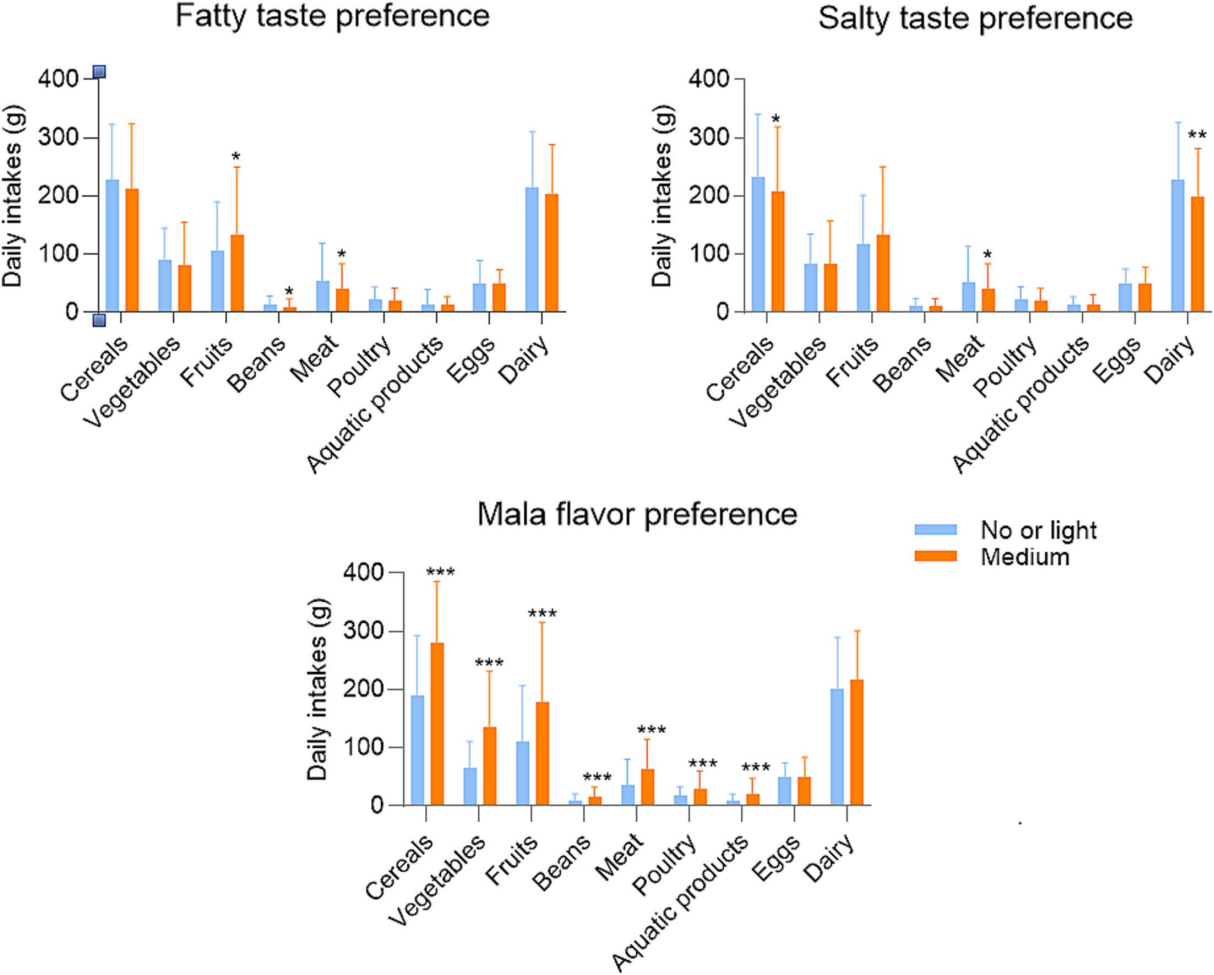
Daily food intakes according to different taste preferences, *p < 0.05, **p < 0.01, ***p < 0.001.

As shown in Figure 1, compared with those with no or light preference, women with a medium preference for Mala flavors had higher intakes of cereals, vegetables, fruits, beans, meat, poultry, and aquatic products. Most food intakes did not differ significantly between women with different fatty (excepting for fruits, beans, and meat) or salty (excepting for cereals, meat, and dairy) flavor preferences.

**Figure 2:**
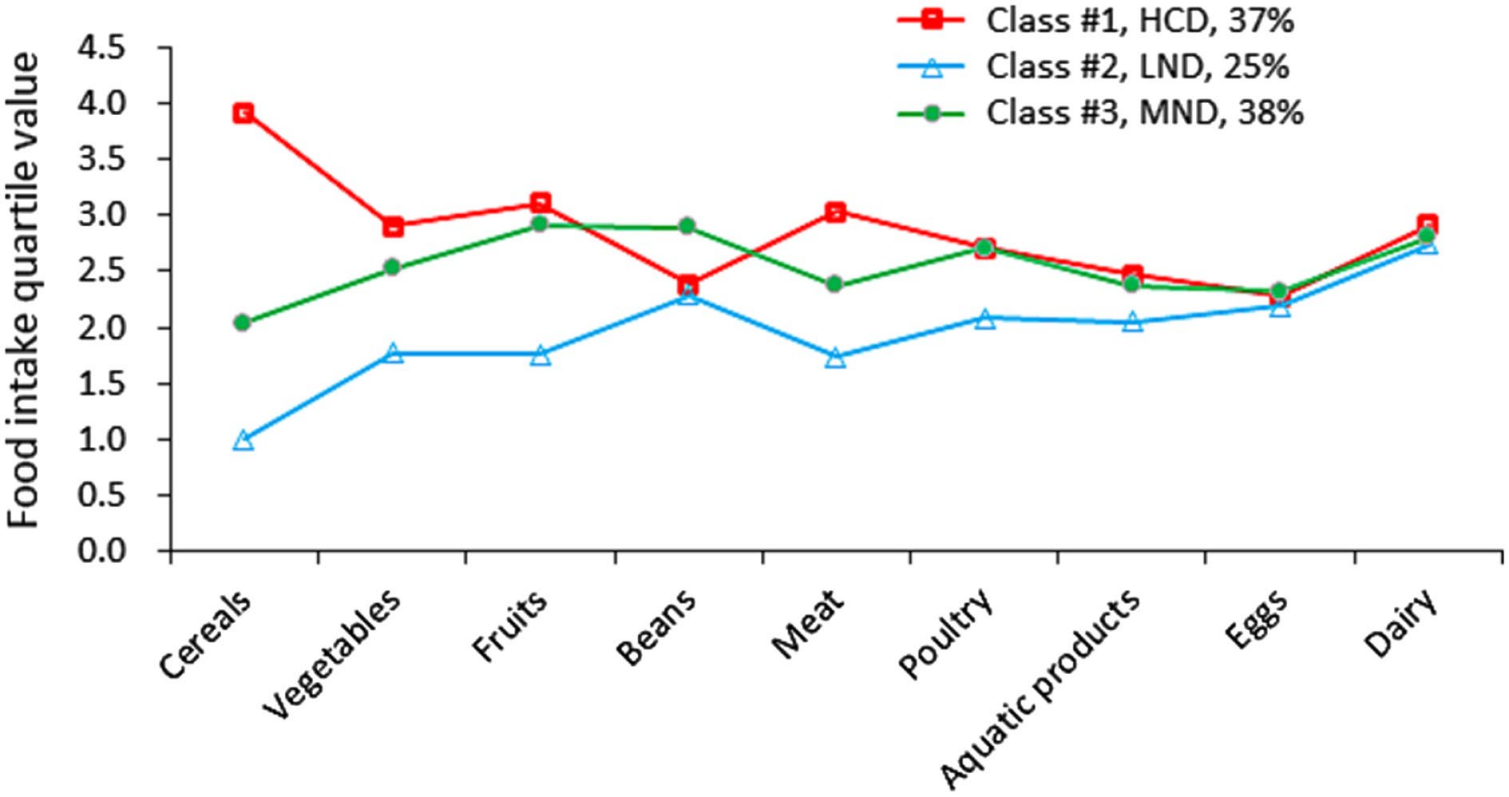
Description of the selected LPA profiles of the daily food intakes. HCD, high carbohydrate dietary; LND, low nutrient dietary; MND, moderate nutrient diet.

The goodness of fit for the models examined indicated that the three-latent-profile solution demonstrated the most favorable fit indicators (Table 3). Class #1, comprising 182 (37%) participants, was characterized by an extremely high intake of cereals and labeled as “high carbohydrate die” (HCD). Class #2, including 124 (25%) participants, exhibited low intake across cereals, vegetables, fruits, meat, poultry, and aquatic products, and was categorized as “low nutrient diet” (LND). Class #3, consisting of 189 (38%) participants, was marked by moderate food intakes and termed “moderate nutrient diet” (MND) (Figure 2).

**Table 3:** Fit indices for the latent profile analysis of daily food intakes in pregnant women in Chongqing.

Model	AIC	BIC	aBIC	Entropy	LMRT p	BLRT p	Class probability
2-class	12190.396	12308.124	12219.251	0.983	<0.001	<0.001	0.62/0.38
3-class	11602.113	11761.886	11641.274	0.979	0.0268	<0.001	0.37/0.25/0.38
4-class	11160.996	11362.815	11210.462	0.981	0.4624	<0.001	0.22/0.35/0.07/0.36

AIC, Akaike Information Criterion; BIC, Bayesian Information Criterion; aBIC, Adjusted Bayesian Information Criterion; BLRT, Bootstrap Likelihood Ratio Test; LMRT, Lo–Mendell–Rubin.

Table 4 indicates that women with a medium preference for fatty or salty flavors had a lower proportion of HCDs and a higher proportion of LNDs and MNDs. Women with a medium preference for Mala flavors had a higher proportion of HCDs and a lower proportion of LNDs.

**Table 4:** Dietary patterns of pregnant women in Chongqing according to different taste preference.

Taste preference	HCD	LND	MND	*p*
Fatty				
No or light	41 (46.6)	14 (15.9)	33 (37.5)	0.041
Medium	141 (34.6)	110 (27.0)	156 (38.3)	
Salty				
No or light	54 (48.6)	22 (19.8)	35 (31.5)	0.013
Medium	128 (33.3)	102 (26.6)	154 (40.1)	
Mala				<0.001
No or light	109 (29.9)	117 (32.1)	139 (38.1)	
Medium	73 (56.2)	7 (5.4)	50 (38.5)	

HCD, high carbohydrate diet; LND, low nutrient diet; MND, moderate nutrient diet.

A flowchart that categorizes the different groups of pregnant women based on the flavor preference and dietary patterns was shown in Supplementary Figure 2.

### 3.3 Relationship between taste preference, dietary pattern, and GWG

**Figure 3:**
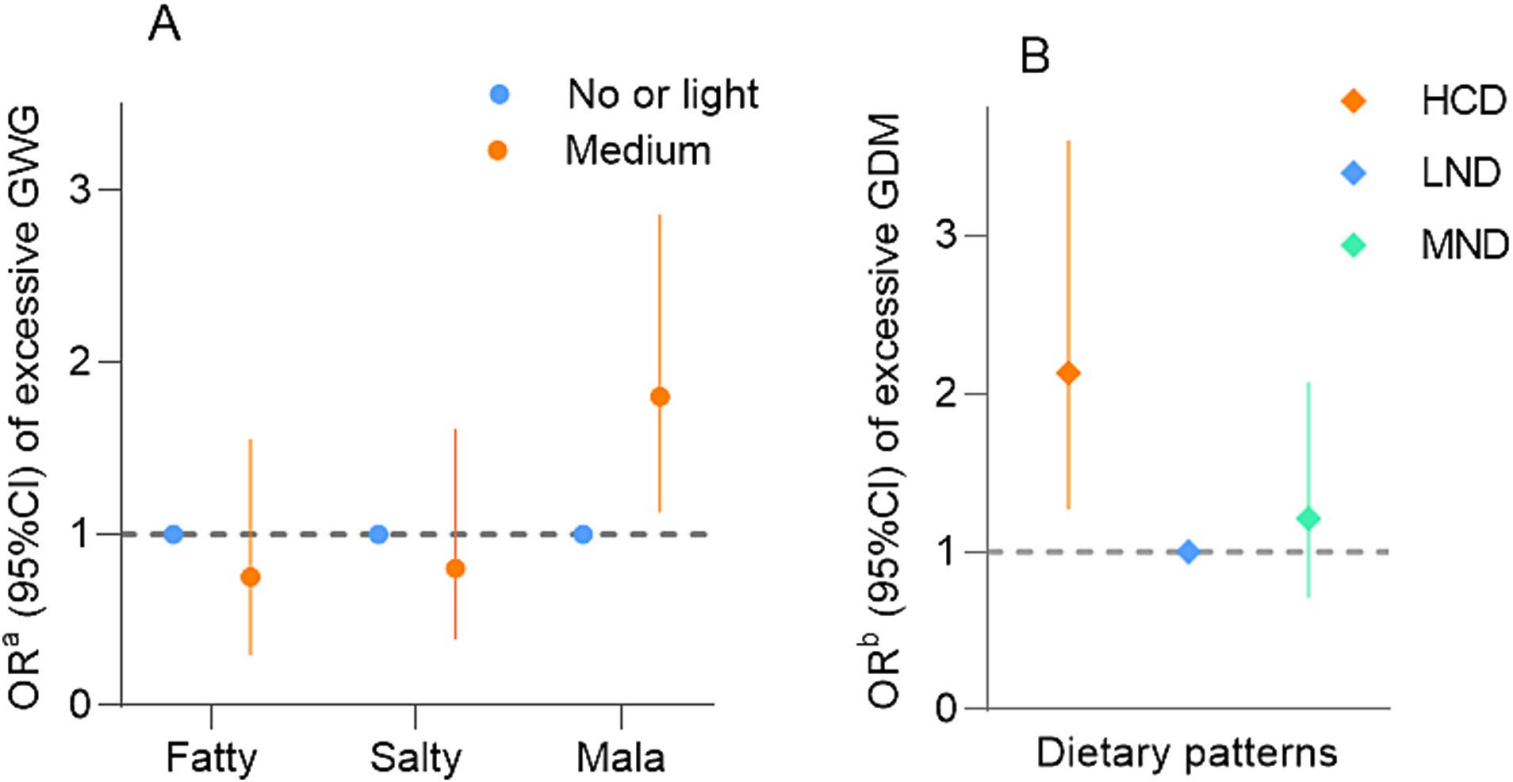
**(A)** The effects of taste preference on excessive gestational weight gain. **(B)** The effects of dietary pattern on excessive GWG. ^a^Adjusted for pre-pregnancy BMI and other taste preferences. ^b^Adjusted for pre-pregnancy BMI. GWG, gestational weight gain; HCD, high carbohydrate dietary; LND, low nutrient dietary; MND, moderate nutrient diet. The effects of taste preference on excessive gestational weight gain.

In Figure 3, women with a medium preference for Mala flavors had a higher risk of excessive GWG (adjusted odds ratio [aOR]: 1.80, 95% confidence interval [CI]: 1.13–2.86) compared with those with no or light preference. Additionally, women with HCDs had a higher risk of excessive GWG (aOR: 2.13, 95%CI: 1.27–3.60) compared with those with LNDs.

**Figure 4:**
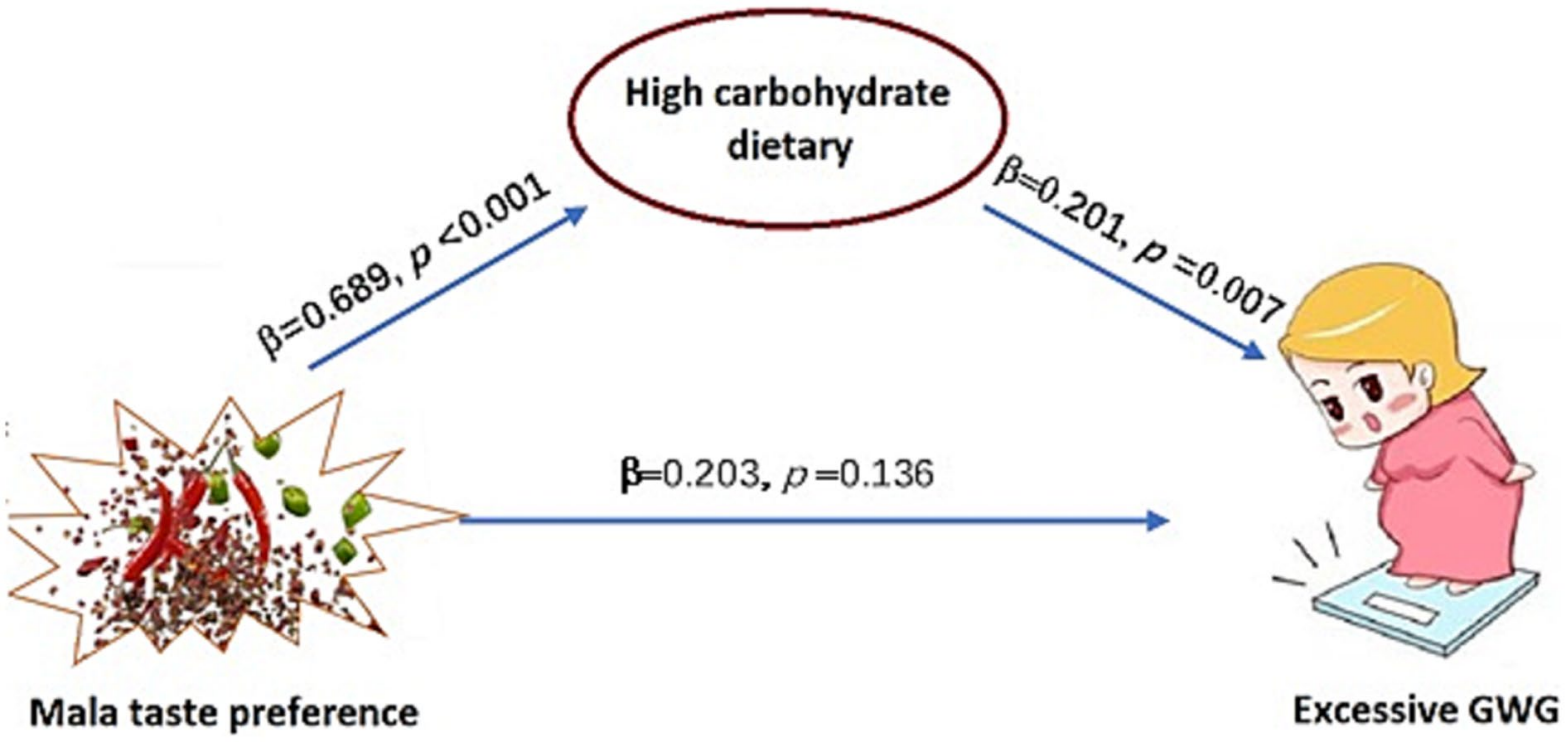
Mediating effect of Mala taste preference on excessive GWG. GWG: Gestational weight gain.

The mediation analysis demonstrated that the model comprising the independent (excessive GWG), dependent (Mala taste preference), and mediator (HCD) variables was within the acceptable fit (comparative fit index = 1.00, Tucker–Lewis index = 1.04, root mean square error of approximation = 0). As shown in Figure 4, the indirect effect of Mala taste preference on excessive GWG mediated by HCDs was significant (*β* = 0.138, *p* = 0.019), while the direct effect was insignificant (*β* = 0. 203, *p* = 0.136).

## 4 Discussion

GWG surpassing the recommended levels in approximately half of pregnancies is often attributed to unhealthy lifestyles and unbalanced diets (26). Excessive GWG is linked to an increased risk of adverse maternal and neonatal outcomes (27, 28). Data from China indicate that 30–50% of women experience excessive GWG (29). Multiple interventions, including diet, physical activity, and combinations, have been proposed to address this issue (30). A meta-analysis showed that dietary interventions are associated with reduced GWG and exert a more significant effect than physical activity alone does (31). Strategies for dietary interventions in weight management should focus on reducing energy intake and consider dietary patterns related to differences in food accessibility, eating behaviors, taste preferences, and cultural background (32). A comprehensive understanding of regional dietary intake elements is crucial for devising targeted dietary interventions to prevent excessive GWG.

In this study, we evaluated dietary intake and taste preference among pregnant women in Chongqing and their impacts on GWG. Our results showed that food intake during pregnancy was notably inadequate, except for cereals, meats/poultries, and eggs, with less than 70% of the recommended minimum intake based on the CBDP being consumed. This is consistent with the 2018 Chongqing Residents’ Health Status report (33). An unbalanced diet heavily laden with carbohydrates has been reported to be associated with weight gain and obesity in Asia (34). This explains why, despite inadequate dietary intake, there are still quite a few cases of excessive GWG in Chongqing (30.3%). We identified three dietary patterns: HCD, LND, and MND by using the LPA method, and found that women adhering to HCD pattern, characterized by a high intake of cereals, had an increased risk of excessive GWG. Our findings align with those of a 2022 Danish study, which demonstrated that a low glycemic index diet was associated with reduced GWG (17). In a similar vein, a 2019 randomized controlled trial established a link between healthy eating practices, including lower carbohydrate intake, and decreased GWG (35). Additionally, a substantial longitudinal study among Korean adults bolsters our conclusions, showing that a diet rich in noodle or rice consumption was inversely related to reductions in waist circumference (36). It is noteworthy that compared to the LND, adhering to a MND pattern did not significantly increase the risk of excessive GWG. This suggests that a balanced diet is a more advantageous approach to ensure the intake of essential nutrients and to manage weight gain during pregnancy effectively.

Taste influences nutritional status and health. A systematic review was carried out in 2023 on the relationship between weight status and the perception of and preference for four common tastes (sweet, salty, fatty, bitter, and sour) by reviewing observational and interventional studies (37). The findings indicated that adults who were overweight and obese exhibited decreased perceptions of the four tastes and that preferences for sweetness and fat increased with weight gain (37). There is no consensus in the literature regarding the effects of food spiciness on obesity. One 2017 prospective study from China suggests that a high intake of spicy foods is positively associated with energy intake but inversely associated with the risk of overweight or obesity (38). A 2023 review of the literature indicated that both pepper extract and capsaicin have shown promising effects in aiding weight reduction (39). However, a meta-analysis of cross-sectional studies in the same year showed that spiciness may adversely affect overweight or obesity (40).

Regarding taste preference during pregnancy, only sugar consumption has been reported in 2019 to contribute to increased GWG and the development of pregnancy complications (41). In the present study, we investigated three taste preferences of pregnant women, namely, fatty, salty, spicy, and Mala flavors, and found that pregnant women who preferred the Mala flavor (but not fatty or salty flavor) had a higher risk of excessive GWG, compared with those who did not. Further analysis showed that the positive effect of consuming foods with a Mala flavor on excessive GWG was mediated by HCD patterns. Unlike the straightforward spiciness found in other regional Chinese cuisines, Sichuan cuisine is distinguished by a unique numbing sensation in its Mala flavor, which is derived from Chinese prickly ash. The numbing compounds in Chinese prickly ash, known as sanshool-based amides, activate sensory neurons in the mouth by targeting TRPV1, a chemically sensitive member of the transient receptor potential (TRP) channels (42, 43). This activation can influence appetite by modulating appetite hormone levels or by regulating gastrointestinal vagal afferent signaling (44). Mala sauce is a staple in Sichuan cooking, used extensively in dishes made with poultry, livestock, fresh fish, beans, and bean products, such as Boiled Meat Slices, Mapo Tofu, Spicy Beef Shreds, Spicy Chicken Slices, and more. Consequently, as our research indicates, the appetite-stimulating effect of Mala flavors lead to increased food intake among pregnant women who prefer this taste, particularly in cereals, and accordingly, a heightened risk of excessive GWG, which is mediated by HCD pattern.

Our study provides an innovative analysis of the effect of preference for the unique Mala taste of Sichuan cuisine on GWG. However, this study has some limitations. First, our FFQ only dealt with food categories, not specific foods, therefore, it was not possible to assess energy and nutrient intake. However, it was effective for collecting the frequency and amount of food category intake and analyzing dietary patterns. Second, the reliability and validity of the FFQ were not evaluated. Nonetheless, this version of the FFQ was an enhancement of the 2014 edition, which had demonstrated good reproducibility and correlations with 24 h dietary recall 2014 (23). And the results of food category intake in our study were consistent with those of the Chongqing Residents’ Health Status Report, which reflects the validity of the FFQ to some extent. Moreover, the FFQ showed good reproducibility. The Spearman and intra-class correlation coefficients between the two FFQ measurements 3 months apart ranged from 0.22 to 0.66 and from 0.22 to 0.60, respectively (Supplementary Table 1). The proportion of women classified into the same or adjacent quartiles by both FFQ measurements ranged from 72 to 85%, whereas the rate of misclassification into opposite quartiles was <9% (Supplementary Table 2). Third, we did not adjust for food intake when analyzing the relationship between taste preferences and GWG, which could potentially introduce bias into the results. However, in the subsequent mediation analysis, dietary patterns extracted on the basis of food category intake were included and adjusted in the model. Another significant limitation is the absence of external validation for our results, which means we cannot confirm the applicability of our findings to other populations. Consequently, it is imperative to approach the interpretation of our findings with caution.

## 5 Conclusion

The current study conducted a thorough evaluation of the dietary intake and taste preferences on GWG in the Southwest China region. Our results indicate that a preference for the Mala flavor, a distinctive characteristic of Sichuan cuisine, is positively associated with GWG, with this relationship being mediated by HCD patterns that feature high glycemic indexes cereals. It is recommended that pregnant women in the Chongqing region consider adjusting their preference for Mala flavors and reducing their consumption of high glycemic index cereals to manage GWG effectively and keep it within a healthy range. However, giving the exploratory nature of this study, caution is advised in interpreting these results. Future research with larger sample sizes is essential to validate our findings and to elucidate the underlying mechanisms, potentially incorporating relevant biomarkers into the analysis.

## Data Availability

The raw data supporting the conclusions of this article will be made available by the authors, without undue reservation.

## References

[1] Godfrey KM Barker DJ. Fetal programming and adult health. Public Health Nutr. (2001) 4:611–24. doi: 10.1079/phn2001145, PMID: 11683554

[2] Goldstein RF Abell SK Ranasinha S Misso M Boyle JA Black MH. Association of gestational weight gain with maternal and infant outcomes: a systematic review and meta-analysis. JAMA. (2017) 317:2207–25. doi: 10.1001/jama.2017.3635, PMID: 28586887 PMC5815056

[3] Simmons D Devlieger R van Assche A Galjaard S Corcoy R Adelantado JM. Association between gestational weight gain, gestational diabetes risk, and obstetric outcomes: a randomized controlled trial post hoc analysis. Nutrients. (2018) 10:1568. doi: 10.3390/nu10111568, PMID: 30360536 PMC6266006

[4] Santos S Voerman E Amiano P Barros H Beilin LJ Bergstrom A. Impact of maternal body mass index and gestational weight gain on pregnancy complications: an individual participant data meta-analysis of European, north American and Australian cohorts. BJOG. (2019) 126:984–95. doi: 10.1111/1471-0528.15661, PMID: 30786138 PMC6554069

[5] Goławski K Giermaziak W Ciebiera M Wojtyła C. Excessive gestational weight gain and pregnancy outcomes. Chin Med J. (2023) 12:3211. doi: 10.3390/jcm12093211, PMID: 37176651 PMC10179218

[6] Chiavaroli V Hopkins SA Biggs JB Rodrigues RO Seneviratne SN Baldi JC. The associations between maternal BMI and gestational weight gain and health outcomes in offspring at age 1 and 7 years. Sci Rep. (2021) 11:20865. doi: 10.1038/s41598-021-99869-7, PMID: 34675369 PMC8531053

[7] Dodd JM Deussen AR Mitchell M Poprzeczny AJ Louise J. Maternal overweight and obesity during pregnancy: strategies to improve outcomes for women, babies, and children. Expert Rev Endocrinol Metab. (2022) 17:343–9. doi: 10.1080/17446651.2022.2094366, PMID: 35768936

[8] Zhang S Li N Li W Wang L Liu E Zhang T. Increased gestational weight gain is associated with a higher risk of offspring adiposity before five years of age: a population-based cohort study. Diabetes Metab Syndr Obes. (2022) 15:2353–63. doi: 10.2147/dmso.S374427, PMID: 35966828 PMC9373997

[9] Koletzko B Godfrey KM Poston L Szajewska H van Goudoever JB de Waard M. Nutrition during pregnancy, lactation and early childhood and its implications for maternal and long-term child health: the early nutrition project recommendations. Ann Nutr Metab. (2019) 74:93–106. doi: 10.1159/000496471, PMID: 30673669 PMC6397768

[10] Marangoni F Cetin I Verduci E Canzone G Giovannini M Scollo P. Maternal diet and nutrient requirements in pregnancy and breastfeeding. An italian consensus document. Nutrients. (2016) 8:629. doi: 10.3390/nu8100629, PMID: 27754423 PMC5084016

[11] Jacques PF Tucker KL. Are dietary patterns useful for understanding the role of diet in chronic disease? Am J Clin Nutr. (2001) 73:1–2. doi: 10.1093/ajcn/73.1.1, PMID: 11124739

[12] Krebs JD Parry-Strong A Braakhuis A Worthington A Merry TL Gearry RB. A Mediterranean dietary pattern intervention does not improve cardiometabolic risk but does improve quality of life and body composition in an Aotearoa New Zealand population at increased cardiometabolic risk: a randomised controlled trial. Diabetes Obes Metab. (2025) 27:368–76. doi: 10.1111/dom.16030, PMID: 39469760

[13] Hussain BM Deierlein AL Talegawkar SA Kanaya AM O'Connor JA Gadgil MD. Concordance between DASH diet and coronary artery calcification: results from the mediators of atherosclerosis in south Asians living in America (MASALA) prospective cohort study. AJPM Focus. (2025) 4:100288. doi: 10.1016/j.focus.2024.100288, PMID: 39587996 PMC11585692

[14] Silva ARC Alicandro G Guandalini VR da Fonseca Grili PP Assumpção PP Barbosa MS. Exploring the link between dietary patterns and gastric adenocarcinoma in Brazil: a mediation analysis. BMC Med. (2024) 22:562. doi: 10.1186/s12916-024-03785-2, PMID: 39609810 PMC11603788

[15] Nordman M Stockmarr A Lassen AD Trolle E. Low-carbon diets across diverse dietary patterns: addressing population heterogeneity under constrained optimization. Sci Total Environ. (2024) 953:176155. doi: 10.1016/j.scitotenv.2024.176155, PMID: 39255932

[16] Mao D Li G Li Y Wang S Zhang M Ma M. Study on the impact of dietary patterns on cardiovascular metabolic comorbidities among adults. Res Sq. (2024). doi: 10.21203/rs.3.rs-4451883/v1, PMID: 38883798 PMC11177970

[17] Geiker NRW Magkos F Zingenberg H Svare J Chabanova E Thomsen HS. A high-protein low-glycemic index diet attenuates gestational weight gain in pregnant women with obesity: the "an optimized programming of healthy children" (APPROACH) randomized controlled trial. Am J Clin Nutr. (2022) 115:970–9. doi: 10.1093/ajcn/nqab405, PMID: 34910089

[18] Cano-Ibáñez N Martínez-Galiano JM Luque-Fernández MA Martín-Peláez S Bueno-Cavanillas A Delgado-Rodríguez M. Maternal dietary patterns during pregnancy and their association with gestational weight gain and nutrient adequacy. Int J Environ Res Public Health. (2020) 17:7908. doi: 10.3390/ijerph17217908, PMID: 33126602 PMC7662940

[19] Li Y Zhou X Zhang Y Zhong C Huang L Chen X. Association of maternal dietary patterns with birth weight and the mediation of gestational weight gain: a prospective birth cohort. Front Nutr. (2021) 8:782011. doi: 10.3389/fnut.2021.782011, PMID: 34901129 PMC8664542

[20] Peinado BRR Frazão DR Bittencourt LO de Souza-Rodrigues RD Vidigal MTC da Silva DT. Is obesity associated with taste alterations? A systematic review. Front. Endocrinol. (Lausanne). (2023) 14:1167119. doi: 10.3389/fendo.2023.1167119, PMID: 37334283 PMC10273260

[21] Luo J Ke J Hou X Li S Luo Q Wu H. Composition, structure and flavor mechanism of numbing substances in Chinese prickly ash in the genus Zanthoxylum: a review. Food Chem. (2022) 373:131454. doi: 10.1016/j.foodchem.2021.131454, PMID: 34731789

[22] Institutes of Medicine; National Research Council Committee to Reexamine. The National Academies Collection: reports funded by National Institutes of Health In: Rasmussen KM Yaktine AL, editors. Weight gain during pregnancy: Reexamining the guidelines. Washington (DC): National Academies Press (2009). 20669500

[23] Li M Halldorsson TI Bjerregaard AA Che Y Mao Y Hu W. Relative validity and reproducibility of a food frequency questionnaire used in pregnant women from a rural area of China. Acta Obstet Gynecol Scand. (2014) 93:1141–9. doi: 10.1111/aogs.12460, PMID: 25053161

[24] Wang J Yang L Li H Li Y Wei B. Dietary selenium intake based on the Chinese food pagoda: the influence of dietary patterns on selenium intake. Nutr J. (2018) 17:50. doi: 10.1186/s12937-018-0358-6, PMID: 29743107 PMC5941689

[25] Verdejo-Garcia A Rossi G Albein-Urios N Lozano OM Diaz-Batanero C. Identifying internalizing transdiagnostic profiles through motivational and cognitive control systems: relations with symptoms, functionality, and quality of life. Compr Psychiatry. (2024) 133:152498. doi: 10.1016/j.comppsych.2024.152498, PMID: 38788615

[26] Goldstein RF Harrison CL Teede HJ. Editorial: the importance of gestational weight gain. Obes Rev. (2020) 21:e13073. doi: 10.1111/obr.13073, PMID: 32608189

[27] Jin C Lin L Han N Zhao Z Liu Z Luo S. Excessive gestational weight gain and the risk of gestational diabetes: comparison of intergrowth-21st standards, IOM recommendations and a local reference. Diabetes Res Clin Pract. (2019) 158:107912. doi: 10.1016/j.diabres.2019.107912, PMID: 31682880

[28] Jiang X Liu M Song Y Mao J Zhou M Ma Z. The Institute of Medicine recommendation for gestational weight gain is probably not optimal among non-American pregnant women: a retrospective study from China. J Matern Fetal Neonatal Med. (2019) 32:1353–8. doi: 10.1080/14767058.2017.1405388, PMID: 29172881

[29] Gou BH Guan HM Bi YX Ding BJ. Gestational diabetes: weight gain during pregnancy and its relationship to pregnancy outcomes. Chin Med J. (2019) 132:154–60. doi: 10.1097/cm9.0000000000000036, PMID: 30614859 PMC6365271

[30] Wu S Jin J Hu KL Wu Y Zhang D. Prevention of gestational diabetes mellitus and gestational weight gain restriction in overweight/obese pregnant women: a systematic review and network meta-analysis. Nutrients. (2022) 14:2383. doi: 10.3390/nu14122383, PMID: 35745114 PMC9231262

[31] Teede HJ Bailey C Moran LJ Bahri Khomami M Enticott J Ranasinha S. Association of antenatal diet and physical activity-based interventions with gestational weight gain and pregnancy outcomes: a systematic review and meta-analysis. JAMA Intern Med. (2022) 182:106–14. doi: 10.1001/jamainternmed.2021.6373, PMID: 34928300 PMC8689430

[32] Smethers AD Rolls BJ. Dietary management of obesity: cornerstones of healthy eating patterns. Med Clin North Am. (2018) 102:107–24. doi: 10.1016/j.mcna.2017.08.009, PMID: 29156179 PMC5726407

[33] Chongqing municipal health commission. (2018) Resident Health Status Report of Chongqing City. Available at: https://wsjkw.cq.gov.cn/zwgk_242/wsjklymsxx/jkfw_266458/gzxx_266460/202009/W020200902369383173212.pdf. (Accessed September 2, 2020).

[34] Mozaffarian D. Dietary and policy priorities for cardiovascular disease, ciabetes, and obesity: a comprehensive review. Circulation. (2016) 133:187–225. doi: 10.1161/circulationaha.115.018585, PMID: 26746178 PMC4814348

[35] Harreiter J Simmons D Desoye G Corcoy R Adelantado JM Devlieger R. Nutritional lifestyle intervention in obese pregnant women, including lower carbohydrate intake, is associated with increased maternal free fatty acids, 3-β-Hydroxybutyrate, and fasting glucose concentrations: a secondary factorial analysis of the European multicenter, randomized controlled DALI lifestyle intervention trial. Diabetes Care. (2019) 42:1380–9. doi: 10.2337/dc19-0418, PMID: 31182492

[36] Park S. Association of a High Healthy Eating Index Diet with long-term visceral fat loss in a large longitudinal study. Nutrients. (2024) 16:534. doi: 10.3390/nu16040534, PMID: 38398858 PMC10892686

[37] Fathi M Javid AZ Mansoori A. Effects of weight change on taste function; a systematic review. Nutr J. (2023) 22:22. doi: 10.1186/s12937-023-00850-z, PMID: 37158889 PMC10165840

[38] Shi Z Riley M Taylor AW Page A. Chilli consumption and the incidence of overweight and obesity in a Chinese adult population. Int J Obes. (2017) 41:1074–9. doi: 10.1038/ijo.2017.88, PMID: 28360431

[39] Sirotkin AV. Peppers and their constituents against obesity. Biol Futur. (2023) 74:247–52. doi: 10.1007/s42977-023-00174-3, PMID: 37493973

[40] Wang M Huang W Xu Y. Effects of spicy food consumption on overweight/obesity, hypertension and blood lipids in China: a meta-analysis of cross-sectional studies. Nutr J. (2023) 22:29. doi: 10.1186/s12937-023-00857-6, PMID: 37291603 PMC10249255

[41] Goran MI Plows JF Ventura EE. Effects of consuming sugars and alternative sweeteners during pregnancy on maternal and child health: evidence for a secondhand sugar effect. Proc Nutr Soc. (2019) 78:262–71. doi: 10.1017/s002966511800263x, PMID: 30501650 PMC7441786

[42] Menozzi-Smarrito C Riera CE Munari C Le Coutre J Robert F. Synthesis and evaluation of new alkylamides derived from alpha-hydroxysanshool, the pungent molecule in szechuan pepper. J Agric Food Chem. (2009) 57:1982–9. doi: 10.1021/jf803067r, PMID: 19209861

[43] He W Liang L Zhang Y. Pungency perception and the interaction with basic taste sensations: an overview. Food Secur. (2023) 12:2317. doi: 10.3390/foods12122317, PMID: 37372528 PMC10296967

[44] Christie S Wittert GA Li H Page AJ. Involvement of TRPV1 channels in energy homeostasis. Front Endocrinol (Lausanne). (2018) 9:420. doi: 10.3389/fendo.2018.00420, PMID: 30108548 PMC6079260

[45] Commission, C. M. H. Resident Health Status Report of Chongqing City. (2018). 2018 p.

