# Supplementary material for: Mala flavor preference increases risk of excessive gestational weight gain mediated by high-carbohydrate dietary patterns in Chongqing, China: an ambispective cohort study
Source: Front Nutr. 2025 Jan 7;11:1464748. doi: 10.3389/fnut.2024.1464748 (PMC11747814; doi:10.3389/fnut.2024.1464748)
Supplement: Supplementary file 1 [file Data_Sheet_1.docx]

**Supplementary Figure 1. Chinese Balanced Dietary Pagoda (CBDP) 2022 version for pregnant women**


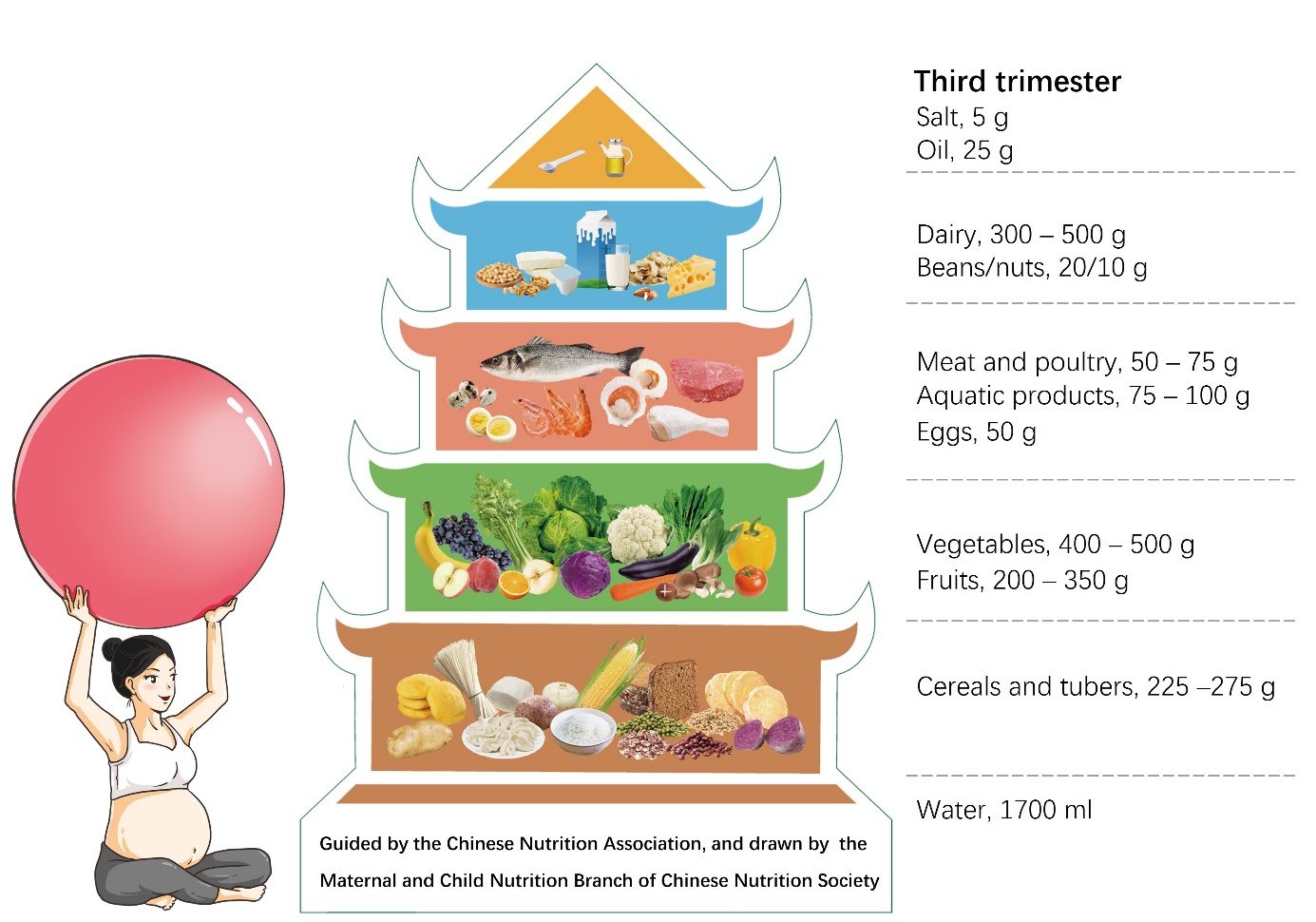


Screened pregnant women who underwent routine prenatal care at the hospital obstetrics department at 24–27 gestational weeks from May 2021 to November 2022 (n=635)

Included (n=500)

Excluded (n=135)

Intended to deliver at a different hospital (n=3)

Received infertility treatment (n=1)

Had multiple pregnancies (n=2)

Had chronic medical disease (n=129)

Analyzed (n=495)

Lost to follow-up (n=3)

Had stillbirth (n=2)

Fatty flavor

Salty flavor

Mala flavor

No/light (n=365)

HCD

109, 29.9%

LCD

117, 32.1%

MND

139, 38.1%

Medium (n=130)

HCD

37, 56.2%

LCD

7, 5.4%

MND

50, 38.5%

No/light (n=111)

HCD

54, 48.6%

LCD

22, 19.8%

MND

35, 31.5%

Medium (n=384)

HCD

128, 33.3%

LCD

102, 26.6%

MND

154, 40.1%

No/light (n=88)

HCD

41, 46.6%

LCD

14, 15.9%

MND

33, 37.5%

Medium (n=407)

HCD

141, 34.6%

LCD

110, 27.0%

MND

156, 38.3%

**Supplementary Figure 2.** Flowchart with the different groups formed according to the flavor preference of the pregnant women and their dietary patterns. HCD: high carbohydrate dietary; LND: low nutrient dietary; MND: moderate nutrient diet.

**Additional table 1**

Spearman correlation coefficients with 95% confidence limits (CL) between the two food frequency questionnaire measurements in pregnant women in Chongqing

| **Food categories** | **Spearman R (95% CL)** | **ICC^1^ R (95% CL)** |
| --- | --- | --- |
| Cereals | 0.66 (0.60, 0.71) | 0.60 (0.54, 0.65) |
| Vegetables | 0.56 (0.50, 0.62) | 0.54 (0.47, 0.60) |
| Fruits | 0.48 (0.41, 0.55) | 0.50 (0.43, 0.56) |
| Beans | 0.33 (0.25, 0.41) | 0.30 (0.22, 0.38) |
| Meats | 0.42 (0.35, 0.50) | 0.49 (0.42, 0.55) |
| Poultry | 0.33 (0.25, 0.41) | 0.30 (0.22, 0.38) |
| Aquatic products | 0.22 (0.13, 0.31) | 0.22 (0.14, 0.30) |
| Eggs | 0.35 (0.28, 0.44) | 0.31 (0.23, 0.39) |
| Dairy | 0.30 (0.22, 0.38) | 0.28 (0.20, 0.36) |

^1^ ICC, intra-class correlation coefficients

**Additional table 2**

Agreement in the quartile distribution of food intake between the two food frequency questionnaire measurements in pregnant women in Chongqing

| **Food categories** | **Same (%)** | **Same or adjacent (%)** | **Opposite (%)** |
| --- | --- | --- | --- |
| Cereals | 65.1 | 76.8 | 3.2 |
| Vegetables | 51.1 | 84.6 | 4.2 |
| Fruits | 47.5 | 84.3 | 5.7 |
| Beans | 50.7 | 74.7 | 8.5 |
| Meats | 47.9 | 81.6 | 3.4 |
| Poultry | 42.8 | 76.3 | 7.1 |
| Aquatic products | 31.3 | 72.1 | 8.5 |
| Eggs | 21.8 | 81.0 | 4.6 |
| Dairy | 47.9 | 80.2 | 2.6 |
